# Tapping-Induced Oxidative Stress Is Associated with Hev b6 Allergen Regulation in *Hevea brasiliensis*

**DOI:** 10.3390/ijms27146110

**Published:** 2026-07-08

**Authors:** Nuntadshaporn Manwang, Benjaporn Noppradit, Sumalee Obchoei, Hansuk Buncherd, Phanthipha Runsaeng

**Affiliations:** 1 Division of Health and Applied Sciences, Faculty of Science, Prince of Songkla University, Songkhla 90110, Thailand; 2 Department of Pharmacognosy and Pharmaceutical Botany, Faculty of Pharmaceutical Sciences, Prince of Songkla University, Songkhla 90110, Thailand; 3 Center of Excellence for Biochemistry, Faculty of Science, Prince of Songkla University, Songkhla 90110, Thailand; 4 Faculty of Medical Technology, Prince of Songkla University, Songkhla 90110, Thailand

**Keywords:** *Hevea brasiliensis*, Hev b6, tapping stress, RNA-seq, plant defense response, laticifer, plant–pathogen interaction, latex allergy

## Abstract

Natural rubber from *Hevea brasiliensis* contains allergenic proteins (Hev b allergens) that can cause latex allergy. This study investigated how repeated mechanical wounding during latex harvesting (tapping) influences allergen regulation in clone RRIT251 using transcriptomic, biochemical, and protein-level analyses. RNA-seq revealed that most genes remained transcriptionally stable, whereas a subset exhibited differential expression trends under tapping conditions. Most *Hev b* allergen genes showed reduced expression, whereas *Hev b6* and *Hev b11* displayed increased expression. qRT-PCR analysis confirmed significant upregulation of these genes, particularly *Hev b6* (approximately 2.3-fold increase). ELISA further demonstrated that Hev b6 accumulated at substantially higher levels in latex (~220 µg/mL) than other Hev b allergens. Gene co-expression and pathway enrichment analyses highlighted plant–pathogen interaction pathways involving reactive oxygen species (ROS), MAPK signaling cascades, and WRKY transcription factors. Biochemical analyses showed that ROS levels were approximately 2.17-fold higher in tapped trees than in untapped controls. Catalase (CAT) activity was also markedly elevated, whereas total superoxide dismutase (T-SOD) activity showed no significant difference between treatments. Together, these findings suggest that tapping-induced mechanical stress is associated with activation of ROS-related defense signaling pathways and prominent accumulation of Hev b6, a defense-associated allergen. This study provides new insights into the relationship between tapping stress, plant defense responses, and latex allergen regulation, and may support future strategies aimed at improving our understanding of allergen regulation while maintaining latex productivity.

## 1. Introduction

Latex allergy represents a significant global public health concern, affecting 1–6% of the general population and up to 12–17% of healthcare workers who experience frequent exposure to natural rubber latex products [1,2]. Natural rubber latex from *Hevea brasiliensis* contains at least 15 distinct allergenic proteins (Hev b1–Hev b15) that trigger IgE-mediated hypersensitivity reactions ranging from contact dermatitis to life-threatening anaphylaxis [3,4]. Among these, Hev b1 (rubber elongation factor), Hev b3 (small rubber particle protein), Hev b5 (acidic latex protein), and Hev b6 (prohevein) are recognized as major allergens responsible for the majority of IgE-mediated sensitization in latex-allergic individuals [5,6]. In particular, Hev b6 exhibits the highest clinical significance, with IgE reactivity detected in 50–90% of affected patients [5,6].

Latex is harvested through tapping, a process involving repeated incisions into the bark tissue to induce latex flow from specialized laticifer cells. This practice constitutes chronic mechanical wounding that imposes continuous physiological stress on rubber trees [7,8]. In plant biology, mechanical wounding triggers defense signaling cascades initiated by damage-associated molecular patterns (DAMPs), which activate pattern-triggered immunity through receptor-mediated recognition [9,10]. A key feature of this response is the rapid generation of reactive oxygen species (ROS), which function as upstream signaling molecules that activate MAPK cascades and WRKY transcription factors to reprogram defense-related gene expression [11,12,13].

Hev b6 represents a unique intersection between plant defense and allergenicity. Its hevein domain confers chitin-binding capacity with potent antifungal activity, classifying it as a pathogenesis-related (PR) protein that contributes to host protection against fungal pathogens [14,15]. This dual role as both a defense protein and a major allergen raises the possibility that tapping-induced defense activation may inadvertently promote allergen accumulation in latex. However, the regulatory mechanisms linking tapping-induced mechanical stress to Hev b6 expression and protein accumulation in *H. brasiliensis* remain poorly understood [16,17].

Despite extensive research on latex biosynthesis and ethylene-mediated responses in rubber trees [18,19,20], a critical knowledge gap persists regarding the mechanistic connection between wound-induced defense signaling and allergen regulation. Previous transcriptomic studies have primarily focused on rubber biosynthesis genes, ethylene signaling, and tapping panel dryness (TPD), a physiological disorder associated with oxidative stress and laticifer dysfunction [21,22,23]. Systematic investigation of how ROS-MAPK-WRKY-associated defense signaling networks is associated with allergen gene expression under tapping stress has not been comprehensively addressed.

The RRIT251 clone is particularly relevant in this context. It is among the most widely cultivated clones in Thailand and Southeast Asia, valued for its high latex yield, broad adaptability across soil types, and strong field resistance to major fungal pathogens including *Phytophthora* leaf fall and *Colletotrichum* secondary leaf fall [24,25]. Its robust field resistance suggests the presence of active defense-associated mechanisms, making RRIT251 an informative biological model for studying how defense-associated pathways intersect with allergen regulation under tapping conditions. Because Hev b6 possesses defense-related functions, RRIT251 provides a useful system for examining potential links between defense activation and allergen regulation under repeated tapping stress. Nevertheless, its allergen expression profile under tapping stress has not been characterized at the molecular level.

Therefore, this study investigated the molecular basis of tapping-induced allergen regulation in *H. brasiliensis* clone RRIT251 through integrated transcriptomic, molecular, protein, and biochemical analyses, with particular emphasis on the ROS-associated defense signaling pathways linked to *Hev b* gene expression.

## 2. Results

### 2.1. Transcriptomic Landscape and Differential Gene Expression

RNA-seq analysis of laticifer-containing bark tissues from *H. brasiliensis* clone RRIT251 included two untapped trees (UT; *n* = 2) and two tapped trees (TP; *n* = 2), resulting in four independent RNA-seq libraries. Sequencing generated a total of 165.2 million raw paired-end reads across all libraries. After quality filtering, 159.6 million clean reads were retained, corresponding to an average of 39.9 million clean read pairs per sample (Table 1). All libraries showed high sequencing quality, with Q20 values ranging from 97.83% to 97.98%, Q30 values ranging from 93.56% to 93.97%, and a mean GC content of 43.85 ± 0.79%.

Clean reads were aligned to the *H. brasiliensis* reference genome (GCF_001654055.1), with total mapping rates ranging from 95.17% to 95.72% and unique mapping rates ranging from 85.77% to 90.33% (Table 1), indicating high-quality sequencing and alignment.

**Figure 1:**
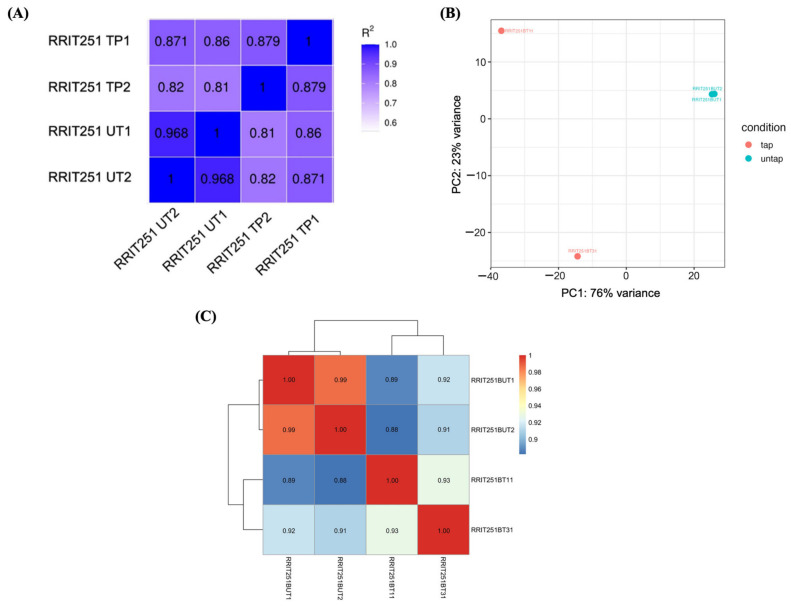
RNA–seq sample quality and clustering analyses. (**A**) Pairwise sample correlation matrix (R^2^). (**B**) Principal component analysis (PCA) of variance-stabilized gene expression data. (**C**) Hierarchical clustering and Pearson correlation heatmap. Samples clustered according to treatment, indicating distinct transcriptomic profiles between untapped (UT) and tapped (TP) trees.

To evaluate the consistency and reproducibility of RNA-seq datasets, pairwise sample correlation analysis, principal component analysis (PCA), and hierarchical clustering were performed (Figure 1). The correlation matrix revealed high similarity among biological replicates, with R^2^ values ranging from 0.81 to 0.968 (Figure 1A). The highest correlation was observed between the untapped biological replicates (UT1 and UT2; R^2^ = 0.968), whereas the lowest correlation was detected between UT1 and TP2 (R^2^ = 0.81). Correlations between samples from different treatment groups ranged from 0.82 to 0.879, indicating distinct transcriptomic profiles between untapped and tapped trees.

Principal component analysis (PCA) of normalized gene expression profiles revealed clear separation between UT and TP samples along the first principal component (PC1), which explained 76% of the total variance, indicating that tapping treatment was the primary source of transcriptomic variation (Figure 1B). The second principal component (PC2) accounted for an additional 23% of the variance. Together, PC1 and PC2 explained approximately 99% of the total variance in the dataset. The two UT biological replicates clustered closely together, reflecting high reproducibility, whereas TP samples showed greater dispersion along PC2, suggesting increased transcriptomic variability under prolonged tapping stress. Nevertheless, all samples remained clearly separated according to treatment, supporting the reliability of subsequent differential expression analyses.

Hierarchical clustering analysis was consistent with the PCA results, with samples grouping according to treatment (Figure 1C). Pearson correlation coefficients were higher within treatment groups (UT1–UT2 = 0.99) than between treatment groups (0.88–0.93), indicating high reproducibility of biological replicates and treatment-associated variation in gene expression. Together, these results support the reliability of the RNA-seq dataset and suggest that long-term tapping stress contributes substantially to transcriptomic variation in rubber tree bark tissues.

**Figure 2:**
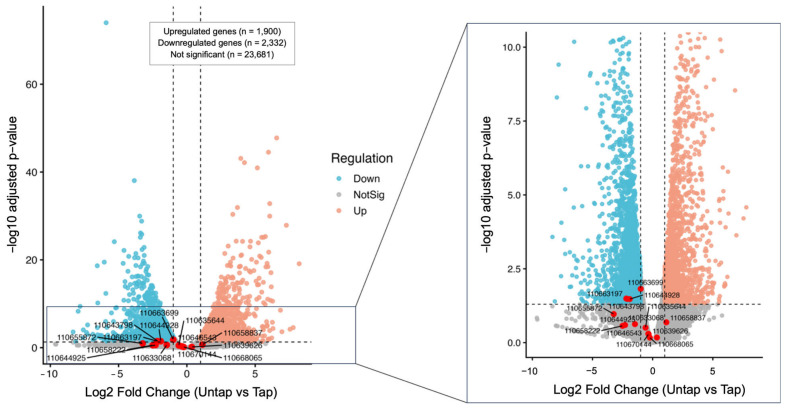
Volcano plot showing differential gene expression between untapped (UT) and tapped (TP) conditions in *H. brasiliensis* clone RRIT251. The x-axis represents log_2_ fold change (log_2_FC), and the y-axis represents −log10(adjusted *p*-value). Orange and blue points indicate significantly upregulated and downregulated genes, respectively (|log_2_FC| ≥ 1 and adjusted *p*-value < 0.05), whereas grey points represent non-significant genes. Horizontal and vertical dashed lines indicate the significance threshold (adjusted *p*-value = 0.05) and fold change threshold (|log_2_FC| = 1), respectively. Red-highlighted points represent *Hev b* allergen-related genes, and the associated numbers correspond to individual *Hev b* gene identifiers. Dashed lines indicate the thresholds for differential expression (|log_2_FC| = 1 and padj = 0.05).

Differential expression analysis identified 27,913 expressed genes, of which 23,681 genes (84.8%) showed no significant expression changes (|log_2_FC| < 1 or padj ≥ 0.05), indicating overall transcriptomic stability between conditions. In contrast, 1900 genes were significantly upregulated and 2332 genes were significantly downregulated in UT samples relative to TP samples (padj < 0.05, |log_2_FC| ≥ 1) (Figure 2). These preliminary RNA-seq results suggest that tapping may induce targeted rather than global transcriptional changes. However, given the limited biological replication (*n* = 2 per group), these transcriptomic observations should be interpreted with caution and were therefore validated using qRT-PCR with additional biological replicates.

### 2.2. Expression Patterns of Hev b Allergen Genes

**Figure 3:**
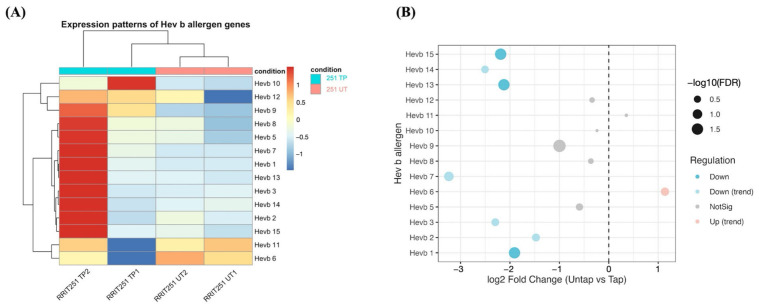
Expression patterns of *Hev b* allergen genes in untapped (UT) and tapped (TP) rubber trees. (**A**) Hierarchical clustering heatmap showing normalized expression levels of 14 *Hev b* allergen genes identified in the transcriptome. Colors represent relative expression levels (Z-score normalized by gene), with red and blue indicating higher and lower expression, respectively. (**B**) Differential expression analysis of *Hev b* allergen genes. The x-axis represents log_2_ fold change (UT vs. TP), and bubble size corresponds to −log10(FDR). Colors indicate expression patterns, including significantly downregulated genes (FDR < 0.05), downregulated trends, non-significant genes, and non-significant upward expression trends.

To investigate the transcriptional regulation of allergen-related genes, the expression profiles of 14 *Hev b* allergen genes (*Hev b1–15*, excluding *Hev b4*, which was not detected in the transcriptome) were analyzed. Hierarchical clustering of normalized expression values revealed distinct expression patterns between UT and TP samples (Figure 3A). Most allergen genes showed lower expression levels in TP samples, whereas *Hev b6* and *Hev b11* displayed relatively higher expression compared with the overall expression trend.

Differential expression analysis identified three significantly downregulated allergen genes following tapping treatment (Figure 3B). *Hev b1* (log2FC = −1.91), *Hev b13* (log2FC = −2.14), and *Hev b15* (log2FC = −1.78) exhibited significantly reduced expression in TP samples relative to UT controls. Several additional allergens, including *Hev b3*, *Hev b5*, *Hev b7*, *Hev b8*, *Hev b9*, *Hev b10*, and *Hev b14*, showed negative log2FC values but did not reach statistical significance.

In contrast, *Hev b6* exhibited the highest positive fold change (log2FC = +1.14; 2.21-fold), while *Hev b11* showed a moderate increase (log2FC = +0.36; 1.28-fold), although neither gene reached statistical significance in the RNA-seq analysis. The non-significant upward expression trends of *Hev b6* and *Hev b11* contrasted with the overall downregulation observed for most other *Hev b* genes. Overall, the *Hev b* genes could be classified into three expression patterns: (i) significantly downregulated genes (*Hev b1*, *Hev b13,* and *Hev b15*), (ii) genes exhibiting non-significant decreases in expression (*Hev b2*, *Hev b3*, *Hev b5*, *Hev b7*, *Hev b8*, *Hev b9*, *Hev b10*, *Hev b12*, and *Hev b14*), and (iii) genes showing non-significant upward expression trends (*Hev b6* and *Hev b11*) (Figure 3B). These RNA-seq expression profiles were subsequently validated by qRT-PCR analysis (Figure 5A).

### 2.3. Functional Enrichment Analysis

**Figure 4:**
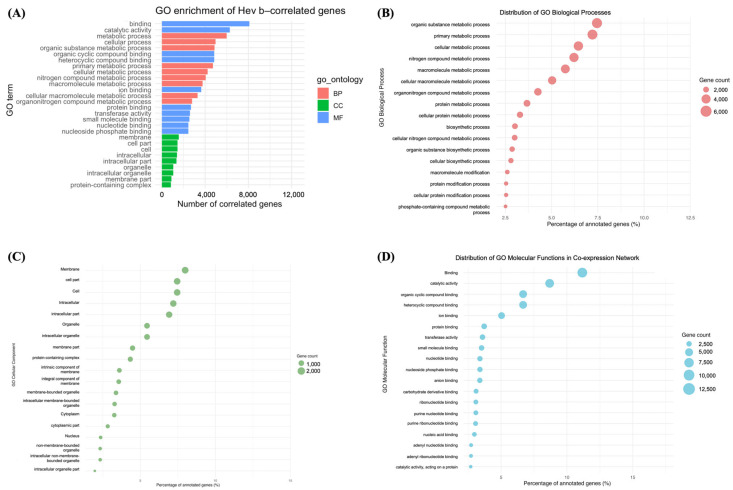
Functional annotation of the Hev b co-expression network. (**A**) Gene Ontology (GO) classification of genes co-expressed with *Hev b* allergen genes (|r| ≥ 0.8). (**B**–**D**) Top enriched GO terms in the biological process (BP), cellular component (CC), and molecular function (MF) categories, respectively. Bubble size indicates gene count, and the x-axis represents the percentage of annotated genes assigned to each GO term.

To further characterize the biological functions associated with Hev b allergen expression, genes showing strong co-expression with *Hev b* genes (|r| ≥ 0.8) were subjected to Gene Ontology (GO) enrichment analysis. A total of 1247 genes were identified as co-expressed with at least one *Hev b* gene and were classified into the three major GO categories: biological process (BP), molecular function (MF), and cellular component (CC) (Figure 4A).

Within the MF category, binding and catalytic activity were the most abundant terms, followed by protein binding, ion binding, nucleotide binding, and transferase activity (Figure 4D), indicating extensive involvement in molecular interactions and enzymatic functions. In the BP category, the co-expressed genes were predominantly associated with metabolic and cellular processes, including primary metabolic process, cellular metabolic process, macromolecule metabolic process, and nitrogen compound metabolic process (Figure 4B). These enrichments suggest substantial transcriptional reprogramming of cellular metabolism in response to tapping. In addition, GO annotations revealed enrichment of stress- and defense-related functions, including oxidation–reduction processes and response to stress.

Within the CC category, enriched terms were mainly associated with membrane, cell part, intracellular structures, organelles, and protein-containing complexes (Figure 4C), indicating that *Hev b*-associated genes are distributed across diverse cellular compartments.

Overall, the GO enrichment analysis suggests that genes co-expressed with Hev b allergens are mainly involved in cellular metabolism, molecular interactions, stress responses, and intracellular organization, supporting a functional link between allergen regulation and tapping-induced stress adaptation.

### 2.4. Quantitative Real-Time PCR (qRT-PCR) Verification

To validate the RNA-seq results and address the limitations associated with low biological replication in the transcriptome analysis, quantitative real-time PCR (qRT-PCR) was performed for 14 selected *Hev b* allergen genes (*Hev b1–3* and *Hev b5–15*) using three biological replicates per condition, with each sample analyzed in technical triplicate.

**Figure 5:**
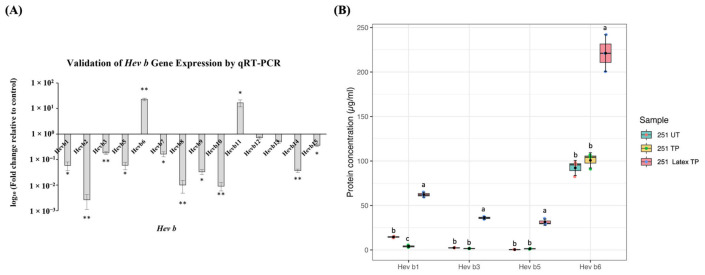
Transcriptional and protein-level validation of Hev b allergens. (**A**) Relative transcript levels of Hev b allergen genes in tapped (TP) bark tissues compared with untapped (UT) controls, as determined by qRT-PCR. Expression values were normalized to the corresponding UT samples. Data represent mean ± SD (*n* = 3 biological replicates). Asterisks indicate significant differences between UT and TP samples (* *p* < 0.05, ** *p* < 0.01). (**B**) Protein concentrations of Hev b1, Hev b3, Hev b5, and Hev b6 in bark tissues (UT and TP) and latex samples collected from tapped (TP) trees, determined by indirect ELISA. Data are presented as mean ± SD (*n* = 3). Different letters indicate significant differences among groups (*p* < 0.05).

Overall, the qRT-PCR results were consistent with the expression trends observed in the transcriptome dataset (Figure 3A), although differences in statistical significance and effect size were observed between the two approaches. Notably, *Hev b6* and *Hev b11* showed significant upregulation by qRT-PCR (Figure 5A), consistent with the increasing trends detected by RNA-seq. *Hev b6* exhibited a 2.34-fold increase by qRT-PCR (*p* < 0.01) compared with a 2.21-fold increase in the transcriptome dataset, whereas *Hev b11* showed a 1.42-fold increase by qRT-PCR (*p* < 0.05) versus a 1.28-fold increase in RNA-seq. Although the fold-change estimates were comparable between methods, these genes were not identified as statistically significant in the RNA-seq analysis.

In addition, several genes, including *Hev b3*, *Hev b5*, *Hev b7*, *Hev b8*, and *Hev b10*, showed significant differential expression by qRT-PCR but not by RNA-seq. Specifically, *Hev b3* (0.68-fold, *p* < 0.05), *Hev b5* (0.71-fold, *p* < 0.05), *Hev b7* (0.65-fold, *p* < 0.01), *Hev b8* (0.59-fold, *p* < 0.01), and *Hev b10* (0.62-fold, *p* < 0.05) were significantly downregulated in TP samples relative to UT samples. In contrast, *Hev b12* showed no significant differences in either method.

Overall, the direction of expression changes was largely consistent between RNA-seq and qRT-PCR analyses, whereas differences in statistical significance likely reflected the greater sensitivity of qRT-PCR for detecting moderate expression changes under limited biological replication. Relative gene expression levels were calculated using the 2^−ΔΔCt^ method [26].

### 2.5. ELISA Verification

To determine whether transcriptional changes were reflected at the protein level, the abundance of Hev b1, Hev b3, Hev b5, and Hev b6 was quantified in bark and latex samples from UT and TP trees using indirect ELISA. The analysis revealed marked differences in allergen abundance among proteins and sample types.

In bark tissue, Hev b6 was the most abundant allergen, with concentrations of approximately 90–100 µg/mL in both UT and TP samples (Figure 5B), and no significant difference was detected between treatments. In contrast, Hev b1 accumulated at substantially lower levels and showed a significant reduction in TP bark compared with UT bark. Hev b3 and Hev b5 were detected at relatively low concentrations (<5 µg/mL) and did not differ significantly between treatments.

In latex samples, Hev b6 also showed the highest abundance, reaching approximately 220 µg/mL which was more than twofold higher than its concentration in bark tissue. Hev b1 was detected at moderate levels (~60 µg/mL), whereas Hev b3 and Hev b5 accumulated at intermediate concentrations of approximately 35 µg/mL and 30 µg/mL, respectively. For all four allergens, protein concentrations were substantially higher in latex than in bark tissues.

Overall, ELISA analysis demonstrated that Hev b6 was the predominant allergen at the protein level in both bark and latex tissues. Although RNA-seq and qRT-PCR analyses revealed only moderate transcriptional induction of Hev b6 following tapping, its consistently high protein abundance suggests that post-transcriptional regulation, protein stability, or preferential accumulation in latex may contribute to its dominant presence in natural rubber latex.

### 2.6. KEGG Pathway Analysis of Hev b6 Co-Expressed Genes

Quantitative PCR and ELISA confirmed that Hev b6 (prohevein) expression and protein accumulation were elevated in laticifer tissues and latex samples. To explore regulatory pathways associated with Hev b6, genes co-expressed with *Hev b6* (|r| ≥ 0.8) were analyzed using the KEGG Pathway Database.

**Figure 6:**
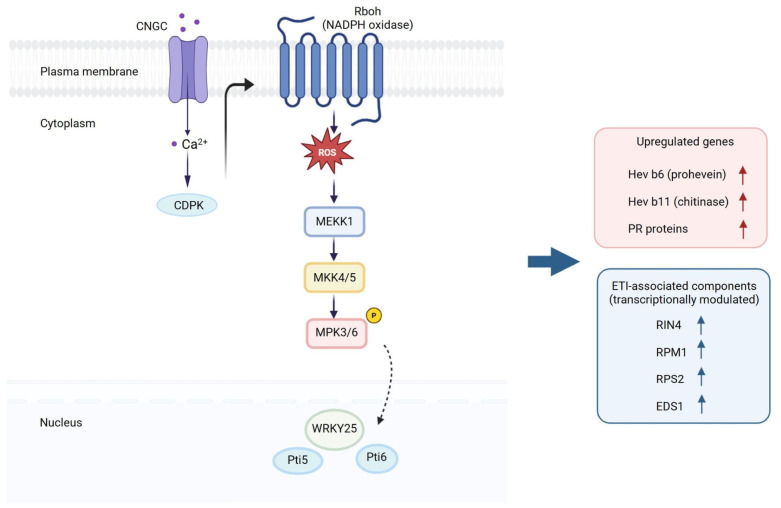
Proposed model of tapping-induced signaling associated with Hev b6 regulation in *H. brasiliensis*. Repeated tapping induces ROS production and Ca^2+^ signaling, leading to activation of MAPK cascades and defense-related transcription factors. These signaling events are associated with the induction of Hev b6, Hev b11, and other defense-related genes. The model was constructed based on RNA-seq, qRT-PCR, ELISA, and KEGG pathway analyses. Solid arrows indicate signaling pathways, red upward arrows indicate gene upregulation, blue upward arrows indicate transcriptional upregulation of ETI-associated components, and the dashed arrow indicates a proposed regulatory interaction.

Mapping these genes onto the plant–pathogen interaction pathway revealed transcriptional modulation of multiple immune signaling components (Figure 6). Key regulators involved in early defense responses, including Rboh, which mediates reactive oxygen species (ROS) production, and components of the MAPK signaling cascade (*MEKK1*, *MKK4/5*, and *MPK4*), were upregulated. For example, *HbRbohA* and *HbRbohB* showed approximately 1.8-fold and 2.1-fold upregulation, respectively, while *HbMPK4* showed approximately 1.7-fold upregulation in TP samples compared with UT samples.

Genes involved in calcium-dependent signaling, including *CDPK*, *CNGC*, and *CaM/CML*, showed variable expression patterns. Several defense-related transcription factors, including *WRKY25*, *Pti5*, and *Pti6*, were induced, whereas *WRKY10* was downregulated. *HbWRKY25* exhibited approximately 2.3-fold upregulation. Downstream defense-related genes such as NHO1 and Rd19 were also upregulated.

In addition, several components associated with effector-triggered immunity (ETI), including *RIN4*, *RPM1*, *RPS2*, and *EDS1*, were upregulated, whereas molecular chaperones involved in resistance protein stability, including *RAR1* and HSP90, were predominantly downregulated.

Overall, KEGG pathway analysis indicated that genes co-expressed with Hev b6 expression were enriched in immune signaling pathways involving ROS production, MAPK signaling, transcriptional regulation, and resistance protein-mediated defense. These results suggest that Hev b6 accumulation is associated with activation of ROS- and defense-related signaling pathways during tapping.

### 2.7. Reactive Oxygen Species and Antioxidant Enzyme Activity Analysis

**Figure 7:**
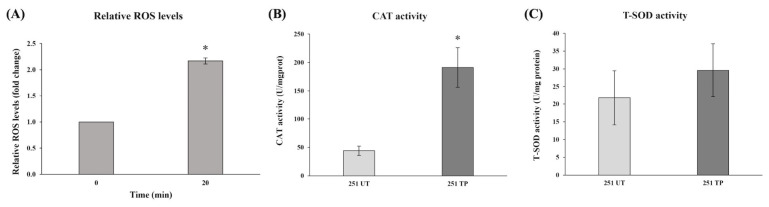
Reactive oxygen species (ROS) accumulation and antioxidant enzyme activities in laticifer-containing bark tissues of rubber trees. (**A**) Relative ROS levels measured at 0 and 20 min after incubation. ROS levels were normalized to the 0 min control. (**B**) Catalase (CAT) activity and (**C**) total superoxide dismutase (T-SOD) activity in untapped (UT) and tapped (TP) samples. Data are presented as mean ± SD (*n* = 3). Asterisks indicate significant differences between treatments (* *p* < 0.05).

To further validate the activation of ROS-related pathways suggested by the KEGG analysis, ROS accumulation and antioxidant enzyme activities were measured in UT and TP trees. ROS levels were significantly higher in TP tissues than in UT tissues, showing an approximately 2.17-fold increase (Figure 7A). This observation is consistent with the transcriptomic data, which revealed upregulation of ROS-producing genes, including members of the Rboh family.

Catalase (CAT) activity was significantly elevated in TP tissues, increasing from approximately 44 to 191 U mg^−1^ protein (4.35-fold; Figure 7B). In contrast, total superoxide dismutase (T-SOD) activity showed no significant difference between TP and UT samples (Figure 7C).

The increased ROS accumulation together with enhanced CAT activity indicates activation of oxidative stress and antioxidant defense responses in tapped rubber trees. These physiological responses are consistent with the enrichment of ROS-related signaling pathways identified by transcriptome and KEGG analyses and support a potential link between tapping-induced oxidative signaling and Hev b6 regulation.

## 3. Discussion

The results of this study suggest that repeated mechanical wounding during latex tapping is associated with activation of defense-related signaling pathways in *H. brasiliensis* and with increased expression and accumulation of Hev b6, a major latex allergen with defense-related functions. The integration of transcriptomic, biochemical, and protein-level analyses provides converging lines of evidence for a potential association between tapping stress and allergen regulation, although the precise mechanistic relationships remain to be fully elucidated.

Because the transcriptomic analysis was based on two biological replicates per condition, the RNA-seq results should be regarded as preliminary transcriptomic evidence. Although this limited replication may reduce statistical power for detecting differentially expressed genes, the subsequent qRT-PCR validation using three independent biological replicates strengthened the reliability of the observed expression patterns, particularly for *Hev b6* and *Hev b11*. Furthermore, the consistency between transcriptomic, protein accumulation, and biochemical analyses provides additional support for the biological relevance of these findings.

Mechanical wounding during tapping likely generates damage-associated molecular patterns (DAMPs), which may be recognized by pattern recognition receptors (PRRs) at the plasma membrane, thereby initiating pattern-triggered immunity (PTI) [10,27]. DAMP perception is commonly associated with a rapid oxidative burst mediated by respiratory burst oxidase homologs (RBOHs), leading to the production of reactive oxygen species (ROS) [11,28]. In the present study, ROS levels were approximately 2.17-fold higher in tapped trees than in untapped controls, supporting the activation of oxidative stress responses following repeated mechanical wounding. This observation is consistent with the transcriptomic upregulation of *HbRbohA* and *HbRbohB*, suggesting enhanced ROS production in tapped tissues.

Consistent with increased ROS accumulation, CAT activity increased approximately 4.35-fold in tapped tissues, whereas T-SOD activity showed no significant difference between treatments. The marked increase in CAT activity suggests that hydrogen peroxide detoxification may represent an important component of the antioxidant response to tapping-induced oxidative stress. Together, these findings suggest activation of ROS-mediated signaling and antioxidant mechanisms that help maintain cellular redox homeostasis in response to tapping. Similar ROS-associated responses have been reported in plant defense systems, where ROS function both as antimicrobial molecules and as signaling intermediates that activate downstream defense pathways [29,30]. The observed induction of Hev b6 is consistent with the activation of ROS- and MAPK-associated defense signaling pathways, which are known regulators of defense gene expression in plants.

Transcriptomic and KEGG pathway analyses further supported the involvement of ROS-mediated signaling pathways. Several components of the MAPK signaling cascade, including *MEKK1*, *MKK4/5*, and *MPK4*, were upregulated in tapped trees. In model plants such as *Arabidopsis*, MAPK cascades are activated by diverse stresses including wounding and pathogen attack, and activated MAPKs subsequently regulate defense-associated transcription factors, including WRKY family proteins [12,31]. In the present study, *WRKY25*, *Pti5*, and *Pti6* were upregulated, whereas *WRKY10* was downregulated. The differential regulation of WRKY transcription factors is consistent with previous observations that WRKY family members may function as either positive or negative regulators of plant defense responses [32,33]. These findings suggest that Hev b6 expression is associated with tapping-induced activation of defense-related transcriptional networks.

Taken together, these findings support a mechanistic framework in which repeated tapping acts as a mechanical stress signal that activates ROS-mediated defense pathways, followed by MAPK signaling and WRKY transcriptional regulation, resulting in increased Hev b6 expression and protein accumulation. This integrative response suggests that Hev b6 is not merely a major latex allergen but also forms part of the defense network activated in laticifer cells during repeated tapping. The coordinated transcriptomic, biochemical, and protein-level responses observed in this study provide evidence linking mechanical stress perception with allergen regulation in *H. brasiliensis*.

A notable finding of this study is the apparent discrepancy between the moderate transcriptional increase in Hev b6 and its exceptionally high protein accumulation in latex and bark tissues. Although qRT-PCR analysis showed approximately 2.3-fold upregulation of *Hev b6* transcripts, ELISA analysis revealed markedly higher Hev b6 protein abundance compared with other Hev b allergens. Notably, the relative magnitude of protein accumulation was substantially greater than the corresponding transcript increase. This observation suggests that regulatory processes beyond transcriptional control may contribute to the high steady-state accumulation of Hev b6 protein in laticifer cells. The structural properties of Hev b6, including its disulfide-rich hevein domain, may enhance protein stability and resistance to proteolytic degradation in the latex environment [14,34]. In addition, efficient targeting through the secretory pathway may facilitate protein accumulation within latex-associated compartments [35,36]. Further studies examining protein turnover, translational efficiency, and subcellular localization would help clarify the mechanisms underlying preferential Hev b6 accumulation.

The activation of defense responses by mechanical wounding is a conserved phenomenon across plant species. In tomato and *Arabidopsis*, wounding activates ROS-, jasmonic acid (JA)-, and MAPK-associated signaling pathways that regulate defense gene expression [37,38,39,40,41]. Previous studies in *H. brasiliensis* have also shown that wounding and ethylene treatment induce genes involved in JA biosynthesis and signaling [42]. Although the primary focus of the present study was the ROS–MAPK–WRKY signaling axis, modulation of JA-related genes was also observed in the transcriptomic dataset. These observations suggest that tapping-induced defense responses in *H. brasiliensis* likely involve crosstalk among multiple signaling pathways, including ROS, MAPK, WRKY, JA, and ethylene signaling networks.

Previous studies have shown that ethylene treatment and mechanical wounding activate jasmonate- and ethylene-responsive defense pathways in Hevea brasiliensis, leading to the induction of genes involved in defense and stress adaptation. Because Hev b6 functions as a defense-related chitin-binding protein, its upregulation observed in the present study is consistent with activation of these signaling pathways. Although jasmonate- or ethylene-mediated regulation was not directly investigated, the transcriptomic evidence together with increased ROS accumulation suggests that ROS-, jasmonate-, and ethylene-dependent signaling pathways may collectively contribute to Hev b6 regulation during repeated tapping. These observations further support the proposed model that Hev b6 is integrated into the broader defense signaling network activated by repeated mechanical wounding.

Interestingly, several components associated with effector-triggered immunity (ETI), including *RIN4*, *RPM1*, *RPS2*, and *EDS1*, were also upregulated in tapped trees. Although ETI is typically associated with pathogen effector recognition [43,44], severe mechanical stress and herbivore damage have also been reported to activate signaling components shared between PTI and ETI pathways [45,46,47]. This interpretation is consistent with the observed upregulation of ETI-associated signaling components (*RIN4*, *RPM1*, *RPS2*, and *EDS1*) in the absence of any intentional pathogen challenge. At the same time, the downregulation of *RAR1* and HSP90, which contribute to resistance protein stability, suggests that tapping may preferentially activate PTI-like defense responses rather than full ETI activation [48,49]. These findings highlight the complexity of immune-related signaling responses induced by repeated tapping stress.

The association between tapping-induced defense signaling and Hev b6 accumulation also has potential implications for latex allergenicity management. Hev b6 is among the most clinically significant latex allergens [5,6], and its elevated accumulation in tapped latex may contribute to allergenic risk in exposed individuals. However, because Hev b6 also possesses defense-related and antifungal properties [14,15], reducing its expression may involve trade-offs with disease resistance and tree health. Agronomic strategies that reduce excessive mechanical stress during tapping, such as optimization of tapping frequency or wound intensity, may help moderate defense pathway activation while maintaining latex productivity [50,51]. In the longer term, improved understanding of the regulatory pathways associated with Hev b6 expression may support breeding or biotechnological approaches aimed at developing rubber clones with lower allergenicity profiles.

Beyond allergenicity, the present findings provide insight into the biology of laticifer cells and the dual role of latex as both a rubber biosynthesis compartment and a defense fluid [16,17,52]. The observation that defense-associated proteins such as Hev b6 are actively regulated in response to tapping supports the view that laticifer cells are dynamically responsive to mechanical stress and that defense functions are integrated into latex physiology.

The activation of ROS-associated defense pathways may also have relevance to tapping panel dryness (TPD), a physiological disorder associated with oxidative stress and cessation of latex flow [21,23,53]. Previous studies have reported altered ROS accumulation and antioxidant enzyme activities in TPD-affected trees [54,55]. Although oxidative stress has been implicated in TPD development, the present study did not directly evaluate TPD symptoms or associated physiological markers. Therefore, the possible relationship between tapping-induced oxidative stress, Hev b6 regulation, and TPD remains hypothetical and warrants further investigation under long-term field conditions. Further longitudinal studies examining oxidative stress markers, TPD-associated physiological traits, and defense gene expression under different tapping regimes would help clarify this relationship.

Beyond the mechanistic insights provided by this study, the present findings also offer several directions for future research and practical applications. Functional validation of key regulatory genes, including MAPK-associated signaling components and WRKY transcription factors, using gene overexpression or gene-silencing approaches will help establish their causal roles in Hev b6 regulation. In addition, comparative transcriptomic analyses across different rubber clones and tapping regimes may identify genetic or physiological factors associated with reduced allergen accumulation while maintaining latex productivity. Such knowledge could contribute to breeding programs for developing low-allergen rubber clones and to the optimization of precision tapping strategies that balance latex yield, tree health, and occupational safety.

## 4. Materials and Methods

### 4.1. Plant Material Collection

Branch bark tissues containing laticifers were collected from 7-year-old *H. brasiliensis* clone RRIT251 trees cultivated in Songkhla Province, Thailand. Two experimental groups were established: untapped trees (Pre-tapping, UT) that had never been subjected to tapping, and trees subjected to regular tapping for 6 months (Post-tapping, TP) following standard commercial tapping practices (S/2 d/3 system: half-spiral cut, tapping every three days). Branches located approximately 50–70 cm above the tapping panel were selected to minimize the influence of immediate local wound responses while allowing the assessment of systemic molecular responses induced by repeated tapping. This sampling strategy was based on the rationale that wound-induced signaling molecules, including reactive oxygen species (ROS), jasmonates, and ethylene, can propagate beyond the immediate tapping site and influence surrounding bark tissues. Samples were collected in the morning to minimize diurnal variation, immediately frozen in liquid nitrogen, and stored at −80 °C until RNA extraction.

### 4.2. Total RNA Extraction

Approximately 0.1–0.3 g of young branch bark tissue from *H. brasiliensis* clone RRIT251 was ground into a fine powder in liquid nitrogen using a mortar and pestle. Total RNA was extracted using the Plant RNA Kit (Omega Bio-tek, Norcross, GA, USA) according to the manufacturer’s instructions. RNA integrity was initially evaluated by 1% agarose gel electrophoresis and visualized using an Alliance Q9 Advanced gel documentation system (Uvitec, Cambridge, UK). RNA concentration and purity were determined spectrophotometrically (NanoDrop 2000, Thermo Fisher Scientific, Waltham, MA, USA) based on A260, A260/A280 (>1.8), and A260/A230 (>2.0) ratios. RNA quality was further assessed using an Agilent 5400 Fragment Analyzer system (Agilent Technologies, Santa Clara, CA, USA). Samples with RNA concentration > 20 ng/µL, total RNA amount > 1.0 µg, and RNA integrity number (RIN) > 7.0 were used for downstream analyses.

### 4.3. RNA-Seq Library Construction, Sequencing and Transcriptome Assembly

RNA sequencing was performed by Novogene Co., Ltd. (Beijing, China) using the Illumina NovaSeq 6000 platform with paired-end 2 × 150 bp sequencing chemistry. Sequencing libraries were constructed from poly(A)-enriched mRNA using oligo(dT) magnetic bead selection, followed by RNA fragmentation, first- and second-strand cDNA synthesis, end repair, A-tailing, adapter ligation, and PCR amplification. Library quality was assessed using the Agilent Bioanalyzer 2100 system prior to sequencing.

Raw sequencing reads were subjected to quality control according to Novogene’s standard data filtering pipeline. Reads were removed if they (i) contained adapter contamination, (ii) had more than 10% undetermined nucleotides (N > 10%), or (iii) contained low-quality bases (Phred score < 5) constituting more than 50% of the read length. Clean reads with Q20 ≥ 96% and Q30 ≥ 91% were retained for all downstream analyses. Sequencing quality metrics for all four RRIT251 libraries are summarized in Table 1.

### 4.4. Transcriptome Alignment and Assembly

Clean reads were aligned to the *H. brasiliensis* reference genome (GCF_001654055.1, BrasilLR_1.0 assembly) using HISAT2 v2.2.1 [56] with default parameters. HISAT2 employs a graph-based alignment strategy that supports splice-aware mapping, enabling accurate alignment of reads spanning exon–exon junctions. Alignment results were visualized using Integrative Genomics Viewer (IGV) v2.8.0. Gene expression levels were quantified as read counts mapped to annotated genomic features using Novogene’s standard quantification pipeline for subsequent differential expression analysis.

Mapping results from all samples were combined and used for reference-guided transcript assembly using StringTie v2.2.1 [57]. Assembled transcripts were compared with reference annotations using Cuffcompare v2.2.1 to identify novel transcripts, novel exons within annotated genes, and refined transcript boundaries. Functional annotation of newly identified transcripts was performed against multiple public databases—including Pfam (protein families), Swiss-Prot (curated protein sequences), Gene Ontology (GO), and Kyoto Encyclopedia of Genes and Genomes (KEGG)—using BLAST+ v2.14.1 searches with an E-value cutoff of 1 × 10^−5^.

### 4.5. Identification of Differentially Expressed Genes (DEGs)

Differential gene expression analysis was performed using the DESeq2 package (v1.32.0) in R (v4.1.0) [58]. Raw read counts were normalized using the median-of-ratios method implemented in DESeq2 to account for differences in library size and RNA composition. Differential expression between UT and TP conditions was assessed using the Wald test, and *p*-values were adjusted for multiple testing using the Benjamini–Hochberg false discovery rate (FDR) method. Genes with an adjusted *p*-value (padj) < 0.05 and an absolute log_2_ fold change (|log_2_FC|) ≥ 1 were considered significantly differentially expressed.

### 4.6. Bioinformatic and Statistical Analysis

Global transcriptional differences between UT and TP samples were visualized using volcano plots generated with the ggplot2 package (v3.3.5) in R [59]. Genes with missing adjusted *p*-values (padj = 0.05) were excluded prior to analysis. Differentially expressed genes (DEGs) were defined as those with padj < 0.05 and |log_2_FC| ≥ 1.

Expression patterns of *Hev b* allergen genes were visualized using dot plots generated within the tidyverse framework (v1.3.1). Gene regulation status was classified based on log_2_FC and adjusted *p*-values into significantly upregulated, significantly downregulated, or non-significant expression categories.

Gene co-expression analysis was performed to identify genes exhibiting coordinated expression patterns with *Hev b* allergen genes. Pearson correlation coefficients were calculated between variance-stabilized expression values (VST; DESeq2) of each detected *Hev b* gene and all expressed genes across samples. Genes showing a strong expression relationship (|r| ≥ 0.8) with at least one *Hev b* gene were defined as co-expressed genes and were subsequently used for functional enrichment analyses.

Gene Ontology (GO) enrichment analysis was conducted using the clusterProfiler package (v4.0.5) [60] and tidyverse packages in R. Co-expressed genes were classified into the Biological Process (BP), Molecular Function (MF), and Cellular Component (CC) categories. Enrichment significance was evaluated using hypergeometric tests followed by Benjamini–Hochberg false discovery rate (FDR) correction (adjusted *p* < 0.05). The most significantly enriched GO terms were identified based on gene counts and enrichment scores for functional interpretation.

Kyoto Encyclopedia of Genes and Genomes (KEGG) pathway enrichment analysis was performed using genes co-expressed with Hev b6. Enriched pathways were identified using KEGG Mapper (https://www.genome.jp/kegg/mapper/; accessed on 26 March 2026) with the *H. brasiliensis* organism code (hbr) and subsequently examined to infer biological processes potentially associated with Hev b6 regulation under tapping-induced stress. Pathway enrichment significance was evaluated using Fisher’s exact test with FDR correction (padj < 0.05).

### 4.7. Quantitative Real-Time PCR (qRT-PCR) Verification

Total RNA was extracted as described in Section 4.2 and treated with DNase I (Thermo Fisher Scientific) to remove genomic DNA contamination prior to first-strand cDNA synthesis using the RevertAid First Strand cDNA Synthesis Kit (Thermo Fisher Scientific) with oligo(dT) primers. The expression levels of 14 *Hev b* allergen genes (*Hev b1–3* and *Hev b5–15*) were analyzed by quantitative real-time PCR (qRT-PCR) using gene-specific primers (Table S1) and *18S rRNA* as the internal reference gene. Reactions were performed using QuantiNova SYBR Green PCR Kit (Qiagen, Hilden, Germany) on a QuantStudio 3 Real-Time PCR System (Applied Biosystems, Foster City, CA, USA). Cycling conditions were: initial denaturation at 95 °C for 2 min, followed by 40 cycles of 95 °C for 5 s, 58 °C for 30 s, 60 °C for 30 s, with a final melt curve analysis from 60 °C to 95 °C. Three biological replicates were analyzed, with each reaction performed in technical triplicate. Relative gene expression levels were calculated using the 2^−ΔΔCt^ method [26]. Statistical significance between UT and TP samples was determined using a two-tailed Student’s *t*-test, with *p* < 0.05 considered statistically significant.

### 4.8. Major Allergenic Protein Determination

#### 4.8.1. Recombinant Protein Production

Recombinant Hev b1, Hev b3, Hev b5, and Hev b6 proteins were produced for subsequent ELISA analysis. The open reading frame (ORF) sequences of each gene were amplified by PCR and cloned into the pET28a(+) expression vector (Novagen, Darmstadt, Germany), which incorporates an N-terminal 6 × His-tag for affinity purification. Recombinant plasmids were transformed into *Escherichia coli* DH5α for plasmid amplification and subsequently introduced into *E. coli* BL21Star(DE3) for protein expression. Protein expression was induced by the addition of 1 mM isopropyl β-D-1-thiogalactopyranoside (IPTG) at 37 °C for 4 h. Bacterial pellets were resuspended in native lysis buffer containing lysozyme and disrupted by sonication. Following centrifugation, the soluble and insoluble protein fractions were collected separately and analyzed by 12% SDS-PAGE to evaluate recombinant protein expression and solubility. Recombinant Hev b5 and Hev b6 proteins were predominantly detected in the soluble fraction and purified under native conditions using a HisTrap FF affinity chromatography column (Cytiva, Uppsala, Sweden). In contrast, recombinant Hev b1 and Hev b3 proteins were mainly recovered in the insoluble fraction, indicating the formation of inclusion bodies. These insoluble proteins were washed and subsequently solubilized in a buffer containing 8 M urea before purification by HisTrap FF affinity chromatography under denaturing conditions. Purified Hev b1 and Hev b3 proteins were then dialyzed against 50 mM Tris–HCl (pH 8.0) at 4 °C for 16 h to remove imidazole and urea. Protein concentrations were determined using the Bradford assay using bovine serum albumin (BSA) as the standard, and the purified proteins were subsequently used for ELISA experiments.

#### 4.8.2. Protein Extraction from Para Rubber Tree and Latex

##### Bark Tissue

Approximately 0.6 g of young rubber tree branch bark tissue was ground in liquid nitrogen and homogenized in extraction buffer (100 mM Tris–HCl pH 8.8, 10 mM EDTA, 0.4% 2-mercaptoethanol, 0.9 M sucrose), followed by phenol extraction using an equal volume of Tris-saturated phenol. Proteins were precipitated with 0.1 M ammonium acetate in methanol at −80 °C overnight. The resulting protein pellets were washed sequentially with methanol and acetone solutions containing 1 mM dithiothreitol (DTT), air-dried, and resuspended in 100 mM Tris–HCl (pH 7.4) containing 0.05% Tween 20. Protein concentration was determined using the Bradford assay, and samples were stored at −20 °C until analysis.

##### Latex

Latex proteins were extracted using an ultrasonic-assisted extraction method. Fresh latex was diluted 1:4 (*v*/*v*) with cold phosphate-buffered saline (PBS, pH 7.4) and centrifuged at 10,000× *g* for 30 min at 4 °C to obtain the water-soluble protein fraction (supernatant). The remaining pellet was resuspended in urea/SDS extraction buffer (8 M urea, 2% SDS, 50 mM Tris–HCl pH 8.0) and subjected to ultrasonic homogenization (3 × 30 s pulses, 40% amplitude) to extract insoluble or poorly soluble proteins. Protein concentrations of both fractions were determined using the Bradford assay prior to downstream analysis.

#### 4.8.3. Enzyme-Linked Immunosorbent Assay (ELISA)

Purified recombinant allergens (rHev b1, rHev b3, rHev b5, and rHev b6; 2 µg/mL each) and protein extracts (1 µg/mL total protein) were coated onto 96-well ELISA plates (Nunc MaxiSorp, Thermo Fisher Scientific) using carbonate–bicarbonate coating buffer (pH 9.6) and incubated at 4 °C overnight. Plates were washed three times with Tris-buffered saline containing 0.05% Tween 20 (TBST) and blocked with 5% skim milk in TBST for 2 h at room temperature. Plates were then incubated with allergen-specific primary antibodies, including anti-Hev b1 (1:5000 dilution), anti-Hev b3 (1:2500 dilution), anti-Hev b5 (1:2500 dilution), and anti-Hev b6 antibodies (1:1000 dilution), for 1 h at 37 °C. After washing with TBST, plates were incubated with HRP-conjugated secondary antibody (goat anti-mouse IgG-HRP, Abcam, ab6789; 1:25,000 dilution) for 1 h at 37 °C. The chromogenic substrate 3,3′,5,5′-tetramethylbenzidine (TMB) was added for signal development at room temperature for 15 min. The reaction was terminated using 0.18 M H_2_SO_4_ stop solution, and absorbance was measured at 450 nm using a SPECTROstar Nano microplate reader (BMG Labtech, Ortenberg, Germany). Allergen concentrations were calculated from standard curves generated using serial dilutions of purified recombinant proteins.

### 4.9. Reactive Oxygen Species and Antioxidant Enzyme Activity Analysis

Reactive oxygen species (ROS) accumulation and antioxidant enzyme activities were determined using bark tissues collected from UT and TP rubber trees. Frozen samples were ground into a fine powder in liquid nitrogen and homogenized in cold extraction buffer (100 mM Tris–HCl pH 8.8) at a ratio of 0.4 g tissue per 1 mL buffer. Homogenates were centrifuged at 12,000 rpm for 30 min at 4 °C, and the resulting supernatants were used for all enzyme and ROS analyses. Total protein content was determined by the Bradford assay and used for activity normalization.

Intracellular ROS levels were measured using the Elabscience^®^ Reactive Oxygen Species (ROS) Fluorometric Assay Kit (Green) (Elabscience Biotechnology, Wuhan, China) according to the manufacturer’s instructions. Extracted supernatants were incubated with 2′,7′-dichlorodihydrofluorescein diacetate (DCFH-DA), and fluorescence intensity was recorded every 1 min for 30 min at 37 °C in the dark using a microplate fluorescence reader (Ex/Em = 488/525 nm). ROS accumulation was calculated as the fold change in relative fluorescence units (RFU) relative to the fluorescence signal at 0 min. For presentation, representative ROS accumulation values at 20 min were used for comparison between treatments.

Catalase (CAT) activity was determined using the Elabscience^®^ Catalase (CAT) Activity Assay Kit according to the manufacturer’s instructions. CAT activity was quantified based on the rate of hydrogen peroxide (H_2_O_2_) decomposition and expressed as U/mg protein, where one unit is defined as the amount of enzyme that decomposes 1 µmol H_2_O_2_ per minute at 25 °C.

Total superoxide dismutase (T-SOD) activity was determined using the Elabscience^®^ Total Superoxide Dismutase (T-SOD) Activity Assay Kit (WST-1 Method) according to the manufacturer’s instructions. T-SOD activity was measured based on the inhibition of WST-1 reduction by superoxide radicals and expressed as U/mg protein. Absorbance values were measured at 450 nm using a microplate reader.

Statistical comparisons between UT and TP groups for enzyme activities were performed using a two-tailed Student’s *t*-test (*p* < 0.05). All assays were performed in triplicate.

## 5. Conclusions

In summary, this study provides preliminary evidence that repeated tapping induces oxidative stress and activates ROS-, MAPK-, and WRKY-associated defense pathways that are associated with increased Hev b6 expression and protein accumulation in *H. brasiliensis*. These findings improve our understanding of the molecular relationship between tapping stress, plant defense responses, and latex allergen regulation. More broadly, this work provides a framework for future studies aimed at identifying key regulatory components controlling allergen accumulation and may support the development of improved tapping practices, low-allergen rubber clones, and sustainable natural rubber production with reduced occupational health risks.

## Figures and Tables

**Table 1:** RNA-seq Quality Metrics for RRIT251 Libraries. Summary of sequencing data quality and alignment statistics for *Hevea brasiliensis* clone RRIT251 RNA-seq libraries.

**Sample ID**	**Condition**	**Library ID**		**Raw Reads**	**Clean Reads**	**Clean Bases**
**RRIT251 UT1**	Untapped (UT)	FRRB220086693-1r		36,580,162	35,091,802	5.26 G
**RRIT251 UT2**	Untapped (UT)	FRRB220086692-1r		45,882,924	43,217,010	6.48 G
**RRIT251 TP1**	Tapped (TP)	FRRB220086701-1r		41,019,466	40,300,884	6.05 G
**RRIT251 TP2**	Tapped (TP)	FRRB220086699-1r		41,716,174	41,039,166	6.16 G
**Mean**				41,299,681.50	39,912,215.50	5.99
**SD**				3,809,285.27	3,443,793.14	0.52
**Sample ID**	**Error Rate (%)**	**Q20 (%)**	**Q30 (%)**	**GC (%)**	**Total Mapping Rate (%)**	**Unique Mapping Rate (%)**
**RRIT251 UT1**	0.03	97.83	93.56	42.91	95.19	90.33
**RRIT251 UT2**	0.03	97.83	93.62	43.48	95.17	87.10
**RRIT251 TP1**	0.03	97.82	93.59	44.38	95.67	85.77
**RRIT251 TP2**	0.03	97.98	93.97	44.61	95.72	86.91
**Mean**	0.03	97.87	93.69	43.85	95.44	87.53
**SD**	-	0.08	0.19	0.79	0.30	1.96

## Data Availability

The data presented in this study are available from the corresponding author upon reasonable request.

## Supplementary Materials

The following supporting information can be downloaded at: https://www.mdpi.com/article/10.3390/ijms27146110/s1.

## Abbreviations

The following abbreviations are used in this manuscript:

BP	Biological Process
BSA	Bovine Serum Albumin
CaM/CML	Calmodulin/Calmodulin-Like Protein
CAT	Catalase
CC	Cellular Component
CDPK	Calcium-Dependent Protein Kinase
CNGC	Cyclic Nucleotide-Gated Channel
DAMPs	Damage-Associated Molecular Patterns
DCFH-DA	2′,7′-Dichlorodihydrofluorescein Diacetate
DEGs	Differentially Expressed Genes
ELISA	Enzyme-Linked Immunosorbent Assay
ETI	Effector-Triggered Immunity
FDR	False Discovery Rate
GO	Gene Ontology
IGV	Integrative Genomics Viewer
JA	Jasmonic Acid
KEGG	Kyoto Encyclopedia of Genes and Genomes
MAPK	Mitogen-Activated Protein Kinase
MF	Molecular Function
PCA	Principal Component Analysis
PR	Pathogenesis-Related
PRRs	Pattern Recognition Receptors
PTI	Pattern-Triggered Immunity
qRT-PCR	Quantitative Real-Time Polymerase Chain Reaction
RAR1	Required for MLA12 Resistance 1
RBOHs	Respiratory Burst Oxidase Homologs
RFU	Relative Fluorescence Units
RIN4	RPM1-Interacting Protein 4
RNA-seq	RNA Sequencing
ROS	Reactive Oxygen Species
RPM1	Resistance to *Pseudomonas syringae* pv. Maculicola 1
RPS2	Resistance to *Pseudomonas syringae* 2
T-SOD	Total Superoxide Dismutase
TP	Tapped (Post-Tapping)
TPD	Tapping Panel Dryness
UT	Untapped (Pre-Tapping)
VST	Variance-Stabilizing Transformation
WRKY	WRKY Transcription Factor (named after conserved WRKYGQK motif)
