# Supplementary material for: Tapping-Induced Oxidative Stress Is Associated with Hev b6 Allergen Regulation in Hevea brasiliensis
Source: Int J Mol Sci. 2026 Jul 8;27(14):6110. doi: 10.3390/ijms27146110 (PMC13411745; doi:10.3390/ijms27146110)
Supplement: Supplementary file 1 [file ijms-27-06110-s001.zip › ijms-4413693-supplementary.pdf]

Table S1. Primer sequences used for qPCR expression analysis of *Hev b* genes.

Gene Symbol	Strand	Sequence (5' → 3')	Amplicon Size (bp)
Hev b1	Forward	CGAAGACAACCAACAAGGGCAG	179
	Reverse	GAGGTACAGCCACGTTCTTCAC	
Hev b2	Forward	TGCAAGGCAACAACCTTCCAC	227
	Reverse	ACTTGACCAGAAGCCACGAAC	
Hev b3	Forward	TCGTGGCACTACTGAGCAGG	194
	Reverse	TCCACATCCAAAACACACCACC	
Hev b5	Forward	AGAAACCGCCGATGCTACAC	183
	Reverse	GCAACAGGCTCAGGCTCAG	
Hev b6	Forward	GCAAGCTCTGCCCCAATAACC	126
	Reverse	CACCACCAACACCTTCGCC	
Hev b7	Forward	GCTGCGACCAATCCTACACTAC	110
	Reverse	ACTAGCCTGCTCTTGCTCTCC	
Hev b8	Forward	CATCGGCCAAGACGGCAG	132
	Reverse	CCACCCAGGTGCAAACCAG	
Hev b9	Forward	GTGGGGTTGGTGCAAACAAAAG	164
	Reverse	TGACATTGAAAGCAGGAACGGG	
Hev b10	Forward	TGCGGCGAAGAGTAAAACGG	141
	Reverse	GTGCGGCAGGGGAAGAAAG	
Hev b11	Forward	TGGAGGTGGTGGTGAAGATGG	106
	Reverse	CTTGGCAGGGCAAGCAGC	
Hev b12	Forward	GCTGCTAAAACCACCGCTGAC	108
	Reverse	TTTACCAGGGAGACCCGCTAC	
Hev b13	Forward	GGGCAGAAAAGGATAGTGCAGG	132
	Reverse	TGTCAACGTGGACGAATGTAGC	
Hev b14	Forward	ACCCCACAAATCAACCTTGCC	119
	Reverse	CCGCCAAGAGAAAGCATCACC	
Hev b15	Forward	CATCGGGACAAACGGGGAC	130
	Reverse	CACGAAAACCAGGACCCTGC	
